# Supplementary material for: Splice-Junction-Based Mapping of Alternative Isoforms in the Human Proteome
Source: Cell Rep. Author manuscript; Available in PMC 2020 Jan 15. (PMC6961840; doi:10.1016/j.celrep.2019.11.026)
Supplement: 2 [file NIHMS1546469-supplement-2.zip › DF1/PXD010154/Spleen/Spleen_4_UGGT1_KDDSFIRPVTF.pdf]

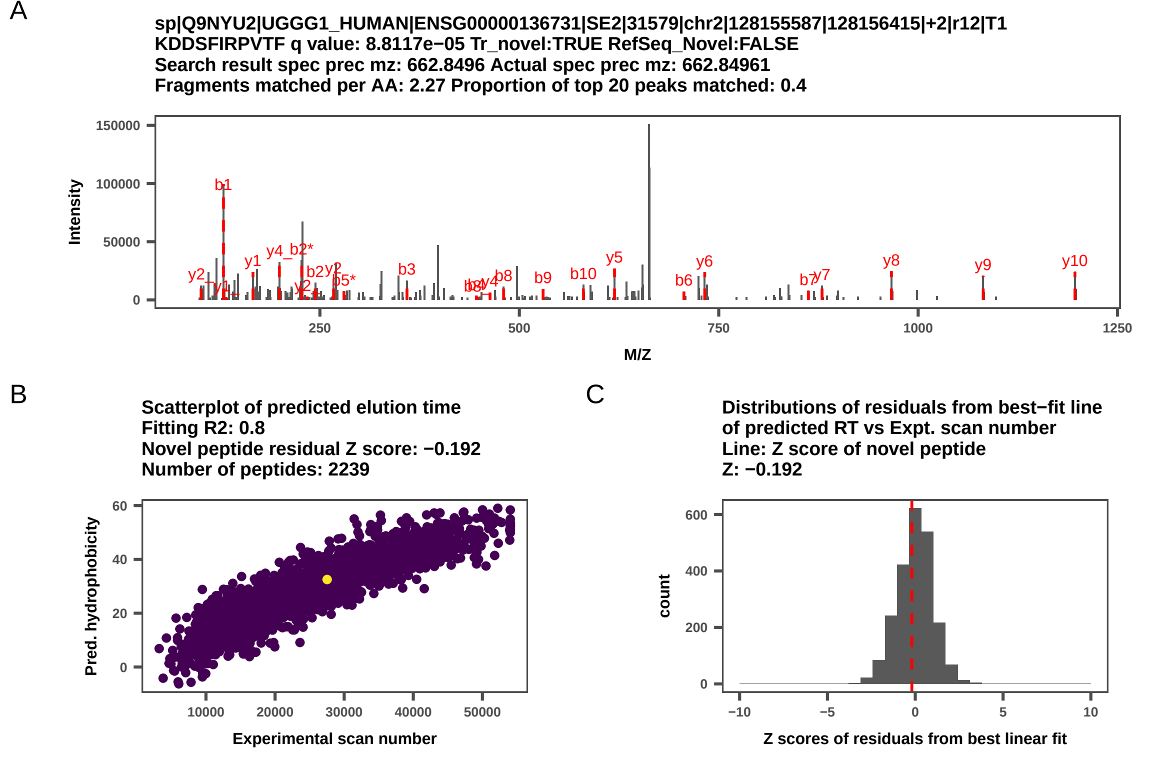
A
sp|Q9NYU2|UGGG1_HUMAN|ENSG00000136731|SE2|31579|chr2|128155587|128156415|+2|r12|T1
KDDSFIRPVTF q value: 8.8117e−05 Tr_novel:TRUE RefSeq_Novel:FALSE
Search result spec prec mz: 662.8496 Actual spec prec mz: 662.84961
Fragments matched per AA: 2.27 Proportion of top 20 peaks matched: 0.4
Intensity
150000
100000
50000
0
b1
y1
y4_b2*
b2y2
b5*
b3
b8
b9
b10
y5
b6
y6
y7
y8
y9
y10
250
500
750
1000
1250
M/Z
B
Scatterplot of predicted elution time
Fitting R2: 0.8
Novel peptide residual Z score: −0.192
Number of peptides: 2239
Pred. hydrophobicity
60
40
20
0
10000
20000
30000
40000
50000
Experimental scan number
C
Distributions of residuals from best−fit line
of predicted RT vs Expt. scan number
Line: Z score of novel peptide
Z: −0.192
count
600
400
200
0
−10
−5
0
5
10
Z scores of residuals from best linear fit
